# Pediatric Fracture Remodeling: From Wolff to Wnt

**DOI:** 10.7759/cureus.78266

**Published:** 2025-01-30

**Authors:** James G Gamble

**Affiliations:** 1 Orthopaedic Surgery, Stanford University School of Medicine, Lucile Packard Children's Hospital, Stanford, USA

**Keywords:** biomechanics, fracture biology, fracture healing, fracture remodeling, fractures in children

## Abstract

Pediatric fracture remodeling is a complex mechanobiological process in which a team of cells, including osteoclasts, osteoblasts, and osteocytes, responds to cytokine and mechanical signals to synthesize new bone in areas of high stress (concavity of a fracture) and remove older redundant bone in areas of low stress (convexity and medullary canal). Piezo1 mechanoreceptors and other pressure-sensitive membrane proteins perceive and convert mechanical strains into intracellular chemical signals. Cytokines are peptides that bind to cell membrane receptors and influence cell functions. Bone morphogenetic proteins and Wnt are the major osteogenic cytokines. Macrophage colony-stimulating factor and receptor activator of nuclear factor κB ligand (RANKL) are the major osteoclastic cytokines. The combination of mechanical stresses and cytokine concentrations stimulates osteoclasts to resorb bone and osteoblasts to make new bone, resulting in remodeling that restores bone strength and structure.

## Introduction

The overall process of fracture healing requires both the union of the fracture and remodeling of the bone. Two of the major determinants of the remodeling phase are the patient’s age and the location of the fracture. A third determinant of remodeling is the orientation of the fracture relative to the plane of motion of the nearest joint. Angulated volar and dorsal wrist fractures align with wrist flexion and extension and will remodel faster and more predictably than fractures with radial or ulnar deviation. Another determinant of remodeling is the growth potential of the nearest physis. A fracture of the proximal humerus has great remodeling potential because the proximal humeral physis contributes ~80% to the overall length of the humerus [1]. A 40-degree angulated mid-diaphyseal humerus fracture in a neonate can completely remodel, but the same angulated fracture in a 12-year-old child will not completely remodel. In general, the younger the patient and the more growth remaining, the greater the remodeling potential [2].

The location of the fracture is important. Metaphyseal fractures remodel more reliably than diaphyseal fractures. The closer to the physis, the greater the remodeling potential [3]. A distal radial fracture that unites with dorsal or volar angulation will remodel by preferential cell proliferation at the concavity to restore relative perpendicularity of the joint to the long axis of the bone [4]. In this article, a case series of remodeled pediatric fractures is presented. 

## Materials and methods

This study was approved by our Institutional Review Board. Specific cases were identified retrospectively from the author's fracture remodeling database to illustrate the principles of pediatric fracture remodeling.

## Results

Case 1

**Figure 1:**
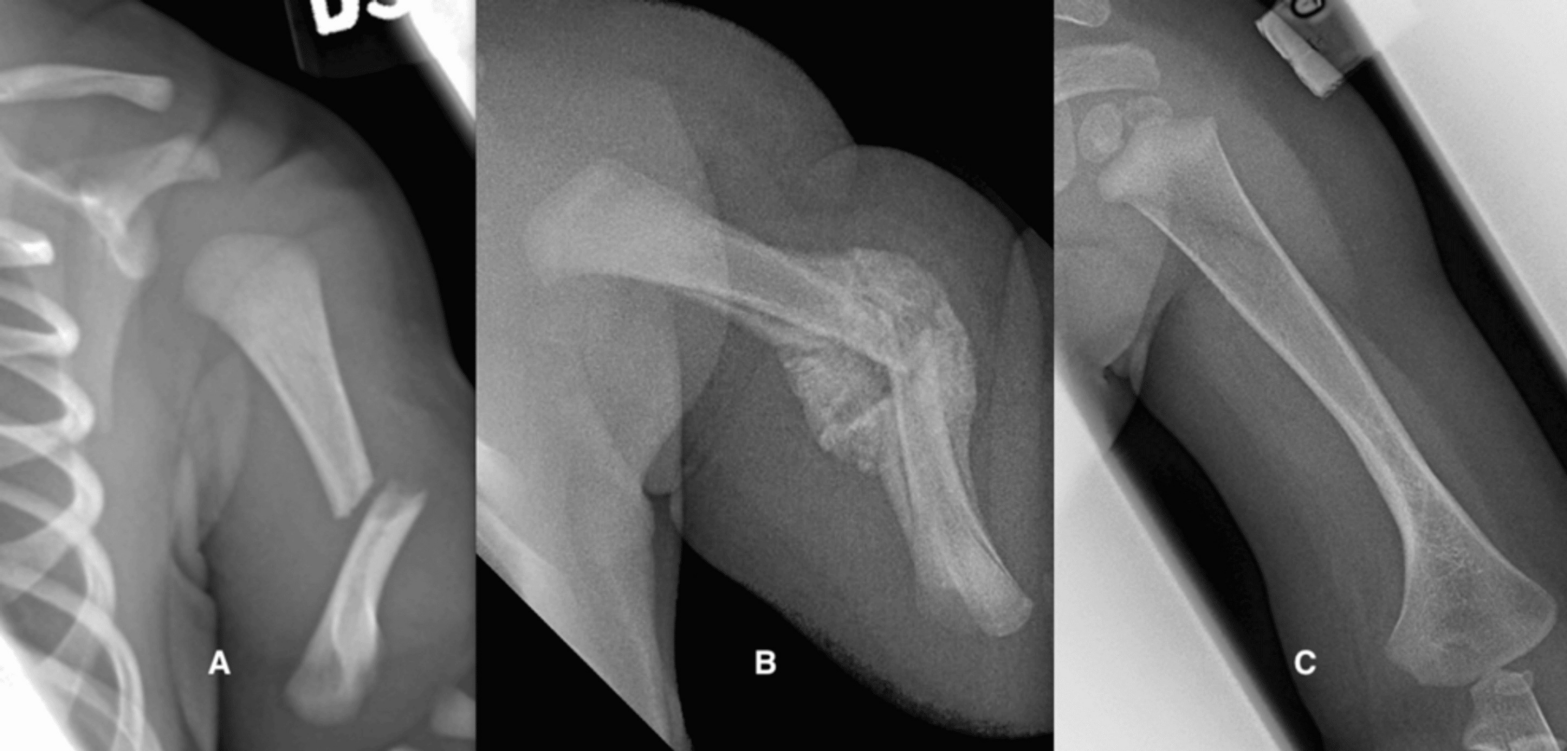
Complete remodeling of a mid-shaft humerus fracture in a neonate, sustained due to shoulder dystocia. A. Initial radiograph showing a 40-degree angulation. B. Abundant hard callus three weeks after injury. C. Normal radiograph one year later.

A full-term male neonate had shoulder dystocia during delivery and sustained a left mid-shaft humeral fracture. Examination disclosed swelling of the humerus and pseudo paralysis with no arm movement during the Moro startle reflex. He was treated with a stockinette Velpeau sling for three weeks. Subsequently, he met all the normal motor milestones, and at the last evaluation when he was one year old, the physical examination and the radiograph were normal (Figure 1).

Case 2

**Figure 2:**
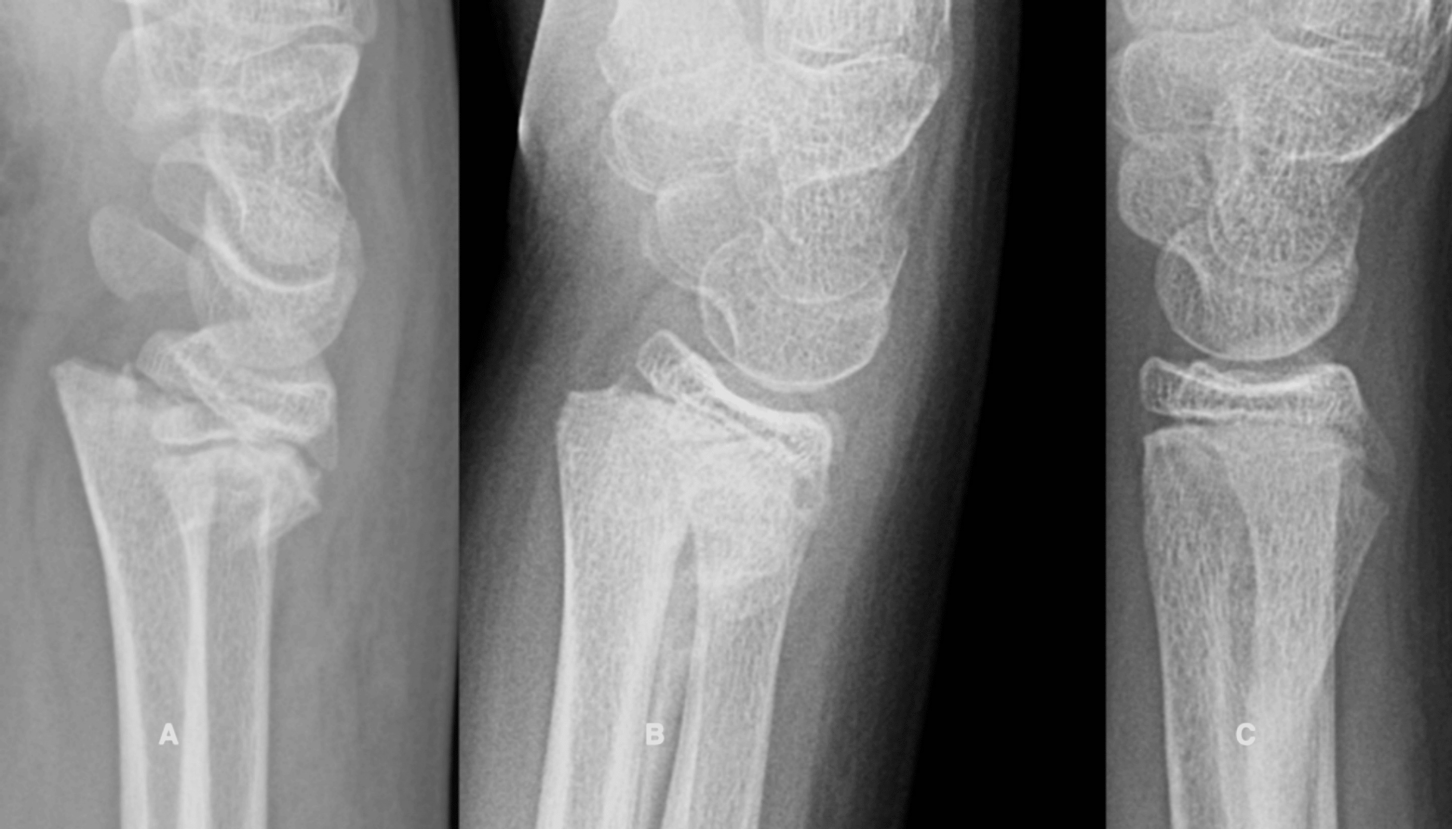
A. 35-degree dorsal angulation of the distal radial physis. B. Six weeks after the fracture, the physis has a 25-degree dorsal angulation. C. Six months after the fracture, the physis and joint are perpendicular to the long axis of the radius.

A ten-year-six-month-old boy fell while running and sustained a right displaced type 2 distal radial fracture with an intact ulna (Figure 2). Attempted closed reduction was unsuccessful and given the possibility of damage to the growth plate from repeated attempts at closed reduction, the position was accepted, given the potential for remodeling if the cells of the growth plate were not damaged. He was treated with a below-elbow cast for one month, followed by the use of a Velcro wrist splint for one month before permitting a gradual return to full activity. At the latest evaluation, he had a full painless range of wrist and elbow motion, and the radiograph was normal.

Case 3

**Figure 3:**
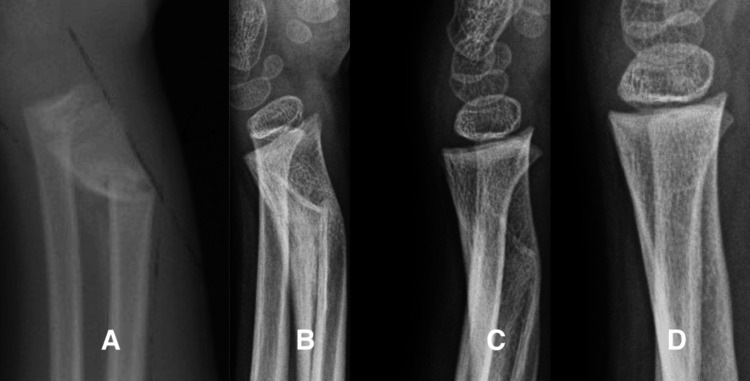
Loss of reduction of a distal radius fracture in a six-year-old boy. A. Two weeks after injury, the distal radius has 40 degrees of dorsal angulation. B. Four months after the fracture, woven bone has filled the concavity, and the sharp angulation at the convexity has smoothed. C. Ten months after the fracture, the remodeled volar cortex touches the remnant of the original dorsal cortex. D.  Eighteen months after the fracture, the metaphysis has completely remodeled and the physis is perpendicular to the long axis of the radius.

A six-year-old boy was seen for a second opinion after a loss of reduction of a right distal radius fracture. Examination two weeks after injury revealed slight swelling of the distal forearm, no motion at the fracture site, and only mild tenderness at the wrist. He was placed in a below-elbow cast for three weeks and then into a Velcro wrist splint for three weeks (Figure 3). At the latest evaluation, he was completely asymptomatic, and the range of motion of his wrist and elbow was normal.

Case 4

**Figure 4:**
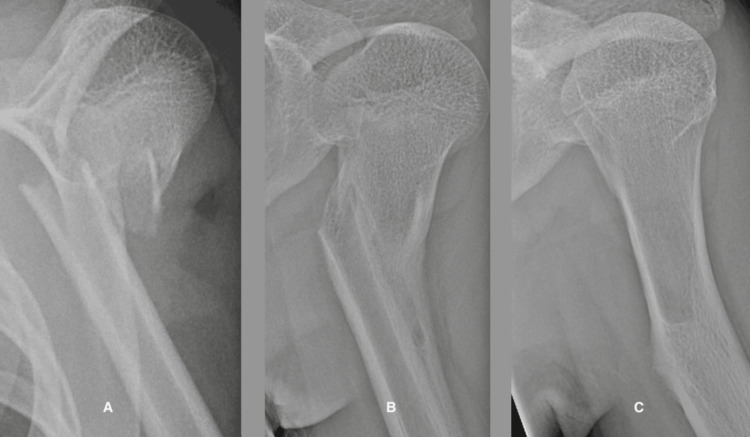
A 10-year-old girl sustained a completely displaced proximal humeral fracture. A. The initial radiograph shows a 100% displacement of the fracture. B. Five months later, she has full painless shoulder motion. C. Two years after the fracture, only a small medial cortical irregularity marks the original medial cortical spike.

A 10-year-old female gymnast fell while performing a floor maneuver and sustained a completely displaced left proximal humerus fracture (Figure 4). She was treated with a sling for three weeks followed by gentle active range of motion exercises and avoidance of weight bearing for two months. At the latest evaluation, she was asymptomatic, had full normal shoulder motion, and had returned to her sport.

Distal radial and distal femoral fractures also have good remodeling potential because their respective physis contribute ~70% to the overall length of the bone [5].

Case 5

**Figure 5:**
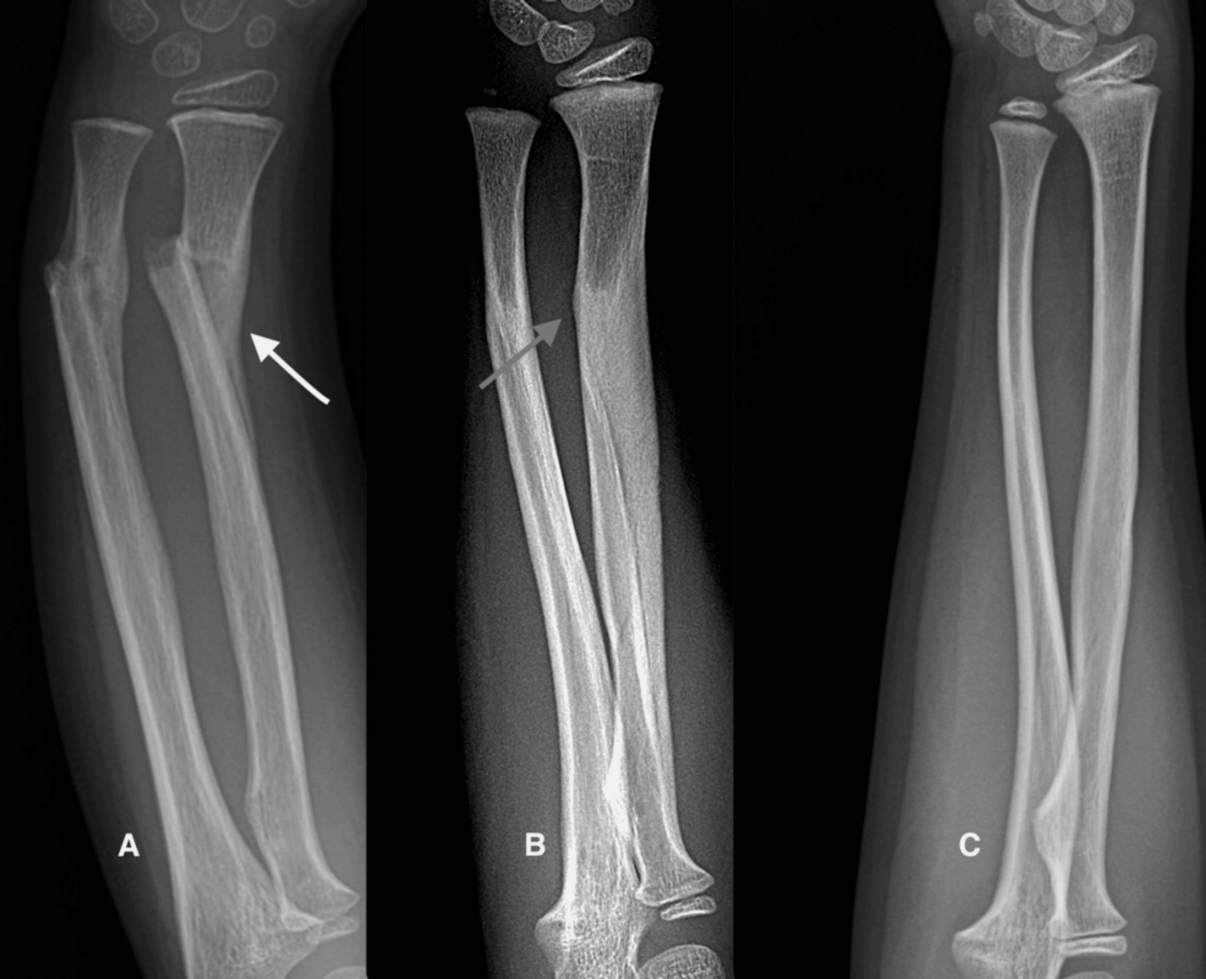
Loss of reduction of a distal forearm fracture with resulting angular deformity in a six-year-old boy. A. Six weeks after the fracture, woven bone fills the concavity (white arrow) but convexity has no new bone.  B. Four months after the fracture, the spike at the convexity is gone (gray arrow), and consolidation has occurred in the concavity.  C. Eight months after the fracture, a normal cortex and medullary canal have been restored due to the mechanical stresses from daily activities.

A six-year-old boy was seen for a second opinion after he had sustained a right distal radius and ulnar fracture when he fell from a jungle gym. He was treated initially with a closed reduction and below-elbow cast immobilization. When we first saw him six weeks after injury, he had 50 degrees of supination and 15 degrees of pronation. We recommended the use of a Velcro wrist splint for three weeks and then gradually return to normal activity. At the latest evaluation, he had returned to full activities and demonstrated a full range of wrist and elbow motion (Figure 5).

Case 6

**Figure 6:**
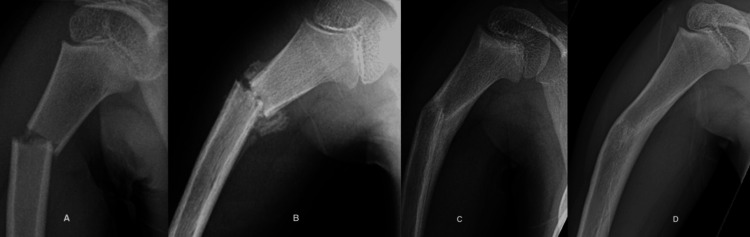
A proximal humeral fracture in a seven-year-old girl showing the phases of fracture healing. A. The initial radiograph shows angulation and displacement of the fracture. B. Three weeks post-injury with a cuff of soft callus.  Note more callus in the convexity. C. Three months after the fracture showing woven bone in the concavity and a smooth cortex on the convexity due to osteoclast activity.  D. One year after the fracture showing restoration of the medullary canal and the cortices.

A seven-year-old girl sustained a right proximal humerus fracture after falling from a bicycle. On examination, she had swelling and tenderness about the right shoulder (Figure 6). She was treated with a sling for a month, at which time she began gradual active motion. At the latest evaluation, she had full shoulder motion, and the contours of the brachium were symmetrical to the un-involved left side.

Case 7

**Figure 7:**
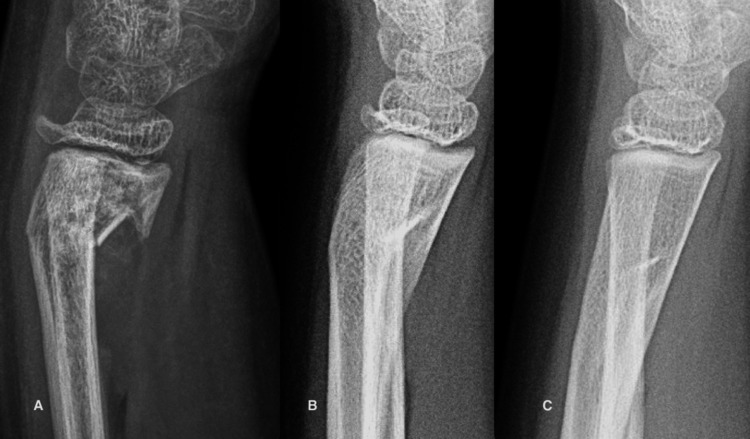
Remodeling of a comminuted distal radial fracture in a 10-year-old boy. A. Early soft callus two weeks after the fracture with flocculent densities in the concavity but none on the convexity.  B. Eight weeks after the fracture, woven bone has united the fracture.  Cortical bone has been resorbed on the convexity. C. Six months after the fracture with restoration of the volar cortex and the medullary canal.  Note the remnant of the volar cortical fragment appearing as a small linear density in the center of the radial shaft.

A 10-year-two-month-old boy presented for a second opinion concerning loss of reduction of a wrist fracture (Figure 7). Two weeks before, he had undergone a closed reduction and casting of a right distal radius fracture. On presentation, he had limited wrist motion but minimal tenderness. He was treated with a below-elbow cast for three more weeks and then a Velcro wrist splint for another three weeks. At the latest evaluation, he had full, painless motion of the wrist and elbow.

Case 8

**Figure 8:**
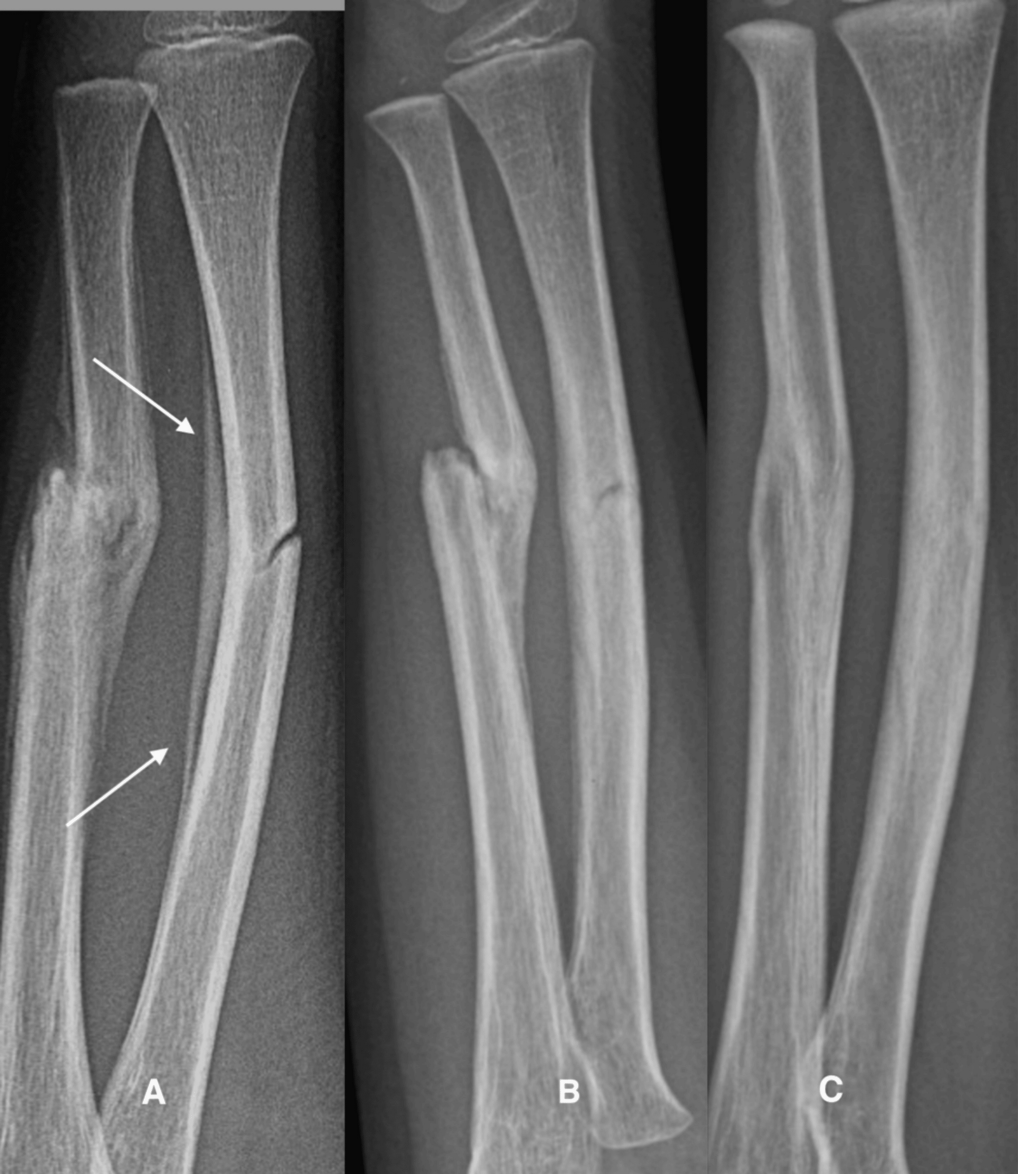
Remodeling of a greenstick fracture of the radius and displaced fracture of the ulna in a six-year-old boy. A: At four weeks, the periosteum over the concavity has been elevated. Osteoblast activity is greatest at the center of the concavity and tapers off proximally and distally (arrows).  The convexity has no evidence of osteoblastic activity. B: At two months, the apex of the radial convexity has smoothed, and osteoblasts have partially restored the cortex. C: At four months, the fractures have united, and remodeling continues at a slower pace to restore the normal cortex and medullary canal.

A six-year-six-month-old boy sustained a right both bones forearm fracture after falling from a play structure. The ulna was displaced and the radius had a green stick fracture. He was placed in an above-elbow cast for one month followed by a below-elbow cast for another month. Two months after the fracture he had no pain, but motion was limited to 50 degrees of pronation and 45 degrees of supination (Figure 8). He was encouraged to do active motion exercises and avoid weight bearing on the arm for another month. At the latest evaluation, he was asymptomatic and demonstrated 90 degrees of supination and 90 degrees of pronation.

Union of pediatric fractures will normally take place in six to eight weeks, but fracture remodeling will continue for many more months until the normal bone structure and strength have been restored.

Case 9

**Figure 9:**
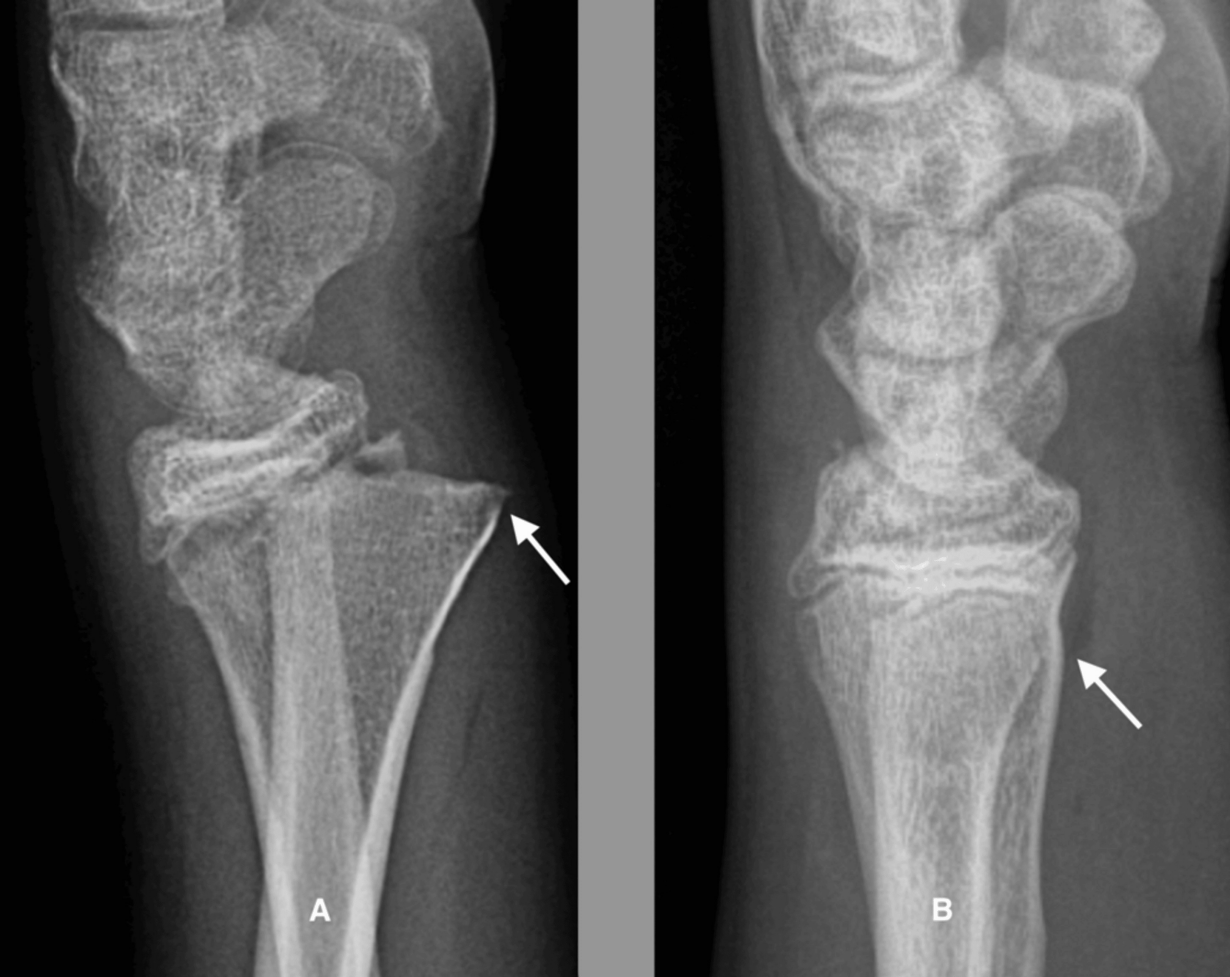
Complete remodeling of a displaced, angulated type II distal radial fracture in a 15-year-old boy. A. Four weeks after the injury, the fracture has united with 25-degree dorsal angulation at the physis and a large volar spike (arrow). B. Two years after the fracture, only a small cortical irregularity remains of the volar spike (arrow) and the physis is perpendicular to the long axis of the radius with complete remodeling.

A 15-year-old boy sustained a left distal radius type 2 fracture that was treated with below-elbow cast immobilization. A closed reduction of the fracture was not attempted due to the possibility of damaging the growth plate (Figure 9). Open reduction was not considered due to the potential of the distal radius to remodel. The cast was used for one month, followed by a Velcro wrist splint for another month. At his last evaluation two years post injury, he had no wrist deformity and a full, painless range of motion.

## Discussion

Clinical observations have shown us what happens during remodeling of pediatric fractures, but to understand why it happens, we must review the basic concepts of bone mechanics and bone biology. 

Bone mechanics

Wolff’s Law

**Figure 10:**
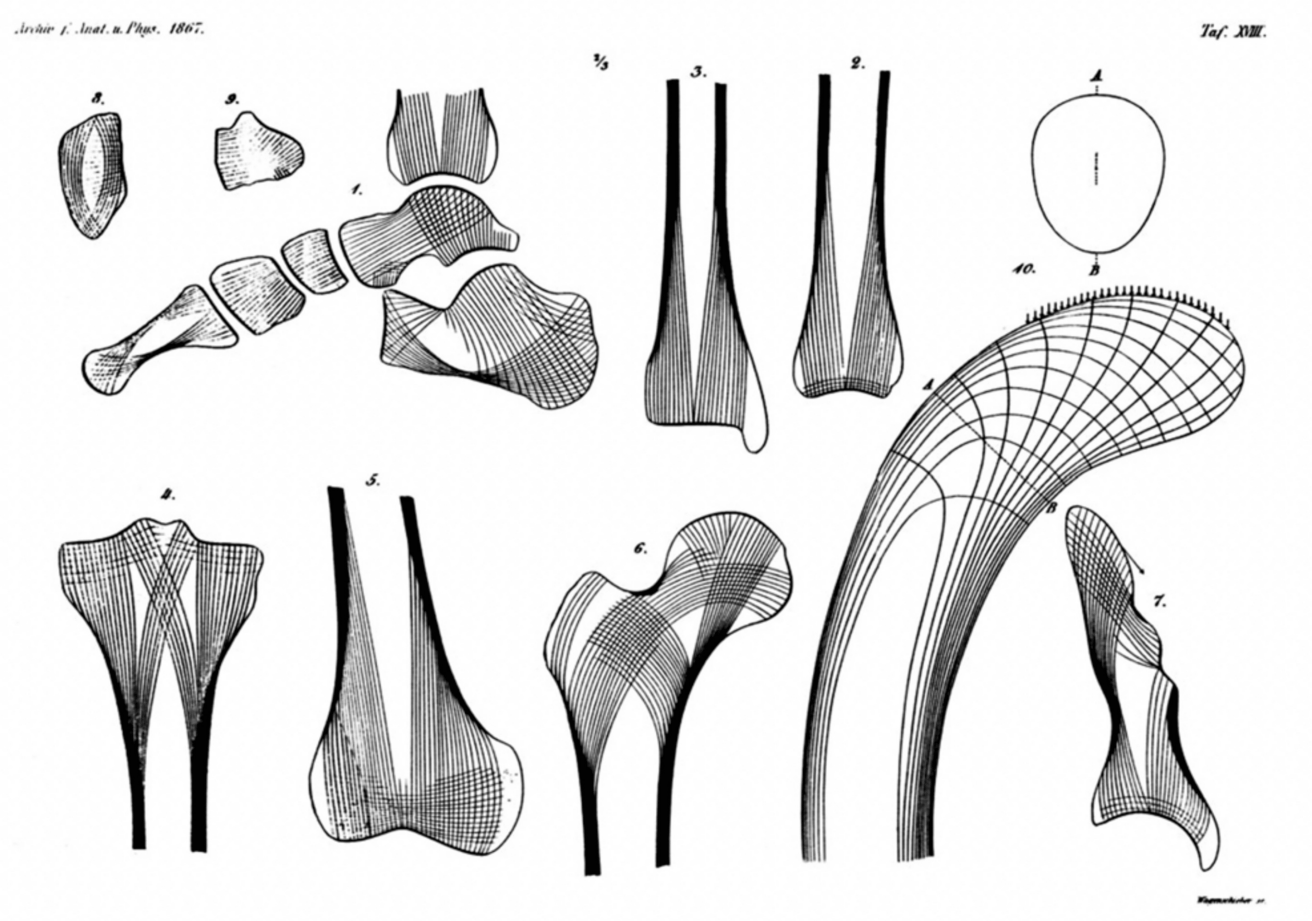
Plate XVII from von Meyer’s 1867 publication. An illustration showing Culmann’s lines of force along the trabecular patterns of multiple bones. Public Domain from [7].

Great ideas seldom appear out of an intellectual vacuum, and so it was with Wolff’s Law. Nineteenth-century anatomists such as Frederick O. Ward (1818-1877), Jean-Baptiste Marc Bourgery (1797-1849), and William Roux (1850-1924) published illustrations on the internal structure of bones, but it was the civil engineer Karl Culmann (1821-1881) who made the connection between mechanical stresses and bone structure. Culmann attended a lecture in Zurich by the anatomist Georg Hermann von Meyer (1815-1892) who showed illustrations of proximal femoral trabecular patterns. Culmann realized that von Meyer’s illustrations resembled the lines of force that he had calculated for a curved Fairbairn crane (Appendix 1) using graphic statics [6]. He shared his idea with von Meyer who realized the importance of the idea and gave Culmann credit for this insight (Figure 10).

Julius Wolff (1836-1902), a professor of surgery in Berlin, had spent decades studying anatomical specimens, and he concluded that bone structure could be explained by mathematical laws. He cited Culmann and von Meyer in his 1892 treatise in which he proposed that bone forms and reshapes in response to bodily function. “Every change in the form and function of the bones, or their function alone, is followed by definite changes in their internal architecture, and equally definite secondary alterations of their external conformation.” While others before Wolff had written about bone structure and function, it was Wolff’s elegant prose that captured the spirit of the process [7]. Thus, we have Wolff’s Law. 

Piezoelectricity

In 1880, the French brothers Pierre (1859-1906) and Jacques Curie (1855-1941) discovered the piezoelectric effect in crystals. The word piezo comes from the Greek meaning to squeeze. Squeezing a crystal causes a voltage, an electrical potential, within the crystal. The younger brother, Pierre, was married to Marie Sokolowski-Curie (1867-1934), and they shared the 1903 Nobel Prize for their research on radiation.

Piezoelectricity remained an interesting physical phenomenon until 1957 when two Japanese researchers, Eiichi Fukada and Iwao Yasuda, discovered that squeezing bones also caused a piezoelectric effect. When stressed by compression, the concavity became electronegative and the convexity electropositive. Bone deposition occurs in the electronegative areas and bone resorption in the positive areas [8].

Orthopedic surgeons C. Andrew L. Bassett (1924-1994) in New York and Carl Brighton (1931-2019) in Philadelphia showed that direct current and even electromagnetic fields could also induce bone formation [9,10]. This led to a lucrative commercial market of devices whose clinical utility is still being investigated.

In 1987, orthopedic surgeon Harold M Frost (1921-2004) published his Mechanostat theory proposing that the quality and frequency of mechanical strain influence bone strength and structure [11]. In areas of high mechanical stress, cells increase bone formation while cells in areas of low stress remove bone. This theory fits well with clinical observations. Frost realized that the forces experienced during daily living such as gravity, muscles pulling on the bone, extremity accelerations, and decelerations all contributed to restoration of normal bone structure after a fracture (Figure 5). 

Mechanotransduction

Wolff’s law and the Mechanostat hypothesis raise another question, “how do the bone cells perceive mechanical stresses?” The answer is mechanotransduction. To understand mechanotransduction, it is helpful to review the microscopic structure of bone.

**Figure 11:**
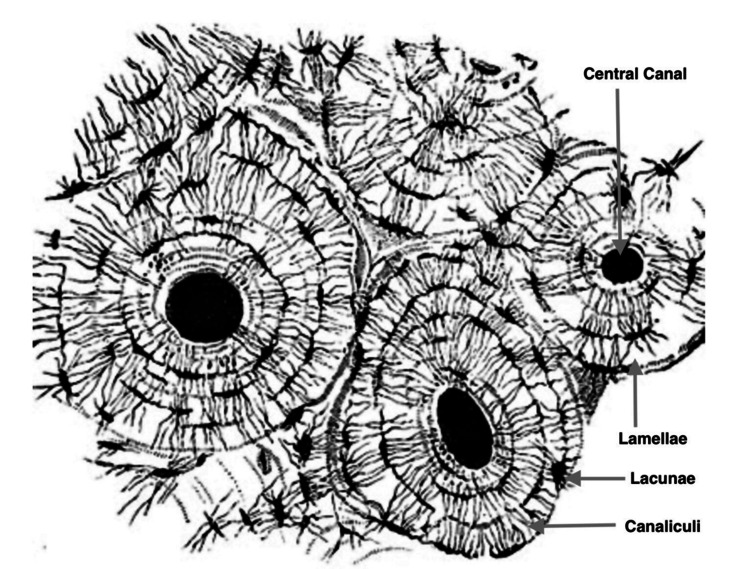
The Haversian system or osteon. Concentric layers of mineralized osteoid, the lamellae, surrounding a central canal containing a blood capillary. Canaliculi that contain osteocyte cell membrane extensions radiate from the lacunae to connect with other lacunae and with the central canal. Modified from [12] Public domain.

Bone is an anisotropic tissue which is stronger in compression than in bending due to its composition and structure. Overall bone composition is roughly 60% mineral, 30% organic matrix, and 10% cells. The basic structural unit of bone is the Haversian system, or the osteon, first described in 1729 by the English physician Clopton Havers (1657-1702). The osteon is a tube-like structure built around a central canal and surrounded by concentric layers of mineralized matrix, the lamellae. Cells reside in small lacunae (Latin for lake) scattered within each lamella. The cells within the lamellae extend 50 to 100 thin membrane projections, dendrites, through microscopic canals, canaliculi, to connect with other cells and with the central canal that contains a blood capillary [12]. It is through these canaliculi that bone cells receive mechanical information (Figure 11).

In 2010, Artem Patapoutian discovered a family of membrane proteins, mechanoreceptors, that convert mechanical strains to intracellular signals. These proteins, called Piezo1 and Piezo2, are transmembrane cation channels that respond to impact by changing their conformation [13]. In 2021, Patapoutian and David Julius shared the Nobel prize for their contributions to our understanding of mechanotransduction. 

Bone cell membranes contain Piezo1 mechanical receptors. These membranes are bathed in a thin layer of extracellular fluid within the lacunocanalicular system. The fluid delivers oxygen and nutrients to the cell and removes carbon dioxide and metabolic waste. This extracellular fluid is also the conduit for mechanical information. Mechanical strains cause minute pressure changes in the extracellular fluid that impact Piezo1, and other pressure-sensitive membrane structures, and cause an almost instantaneous metabolic response in the cells located deep within an osteon [14].

Although our understanding is incomplete, Yasuda, Frost, and Patapoutian, along with an army of other researchers, have given us experimental evidence to help explain Wolff’s idea that form follows function for bone remodeling. 

Bone biology

Cytokines, Signaling, and Cells

Genes that are active during embryogenesis, like homeotic genes, are also active during fracture union and remodeling. Homeotic genes (Appendix 2) encode transcription factors that control body patterns and the individual structure and shape of the bones. During fracture healing, homeotic genes work to ensure that a humeral fracture heals to look like a humerus and not like an amorphous blob of bone, and a femoral fracture heals to look like a femur [15].

Fracture Healing: An Executive Summary

Fracture healing can best be described as occurring in four overlapping phases based on the cellular and matrix preponderance in each phase. The first phase is the inflammatory phase, followed by the fibrovascular-soft callus phase, the hard callus phase, and the remodeling phase [16].

The trauma that resulted in the bone fracture (Figure 6A) also disrupts the periosteum, endosteum, and local blood vessels and soft tissues. The resulting hematoma surrounds the fracture fragments and fills the medullary canal. Plasmin converts fibrinogen to fibrin, and platelets release proinflammatory cytokines into the fibrin clot. Inflammatory cells invade, releasing other cytokines that attract mesenchymal stem cells which organize the fibrin clot into granulation tissue. Devascularized bone cells at the ends of the fragments undergo apoptosis. Special macrophages called osteomacs remove damaged matrix and cellular debris. Chondroblasts and fibroblasts make a fibrocartilaginous soft callus. In this hypoxic environment, calcium and phosphates precipitate out of the extracellular fluid (Figure 6B). The precipitated mineral restricts oxygen and nutrient diffusion resulting in apoptosis of the chondrocytes. Other cytokines recruit capillaries to grow into the area and deliver nutrients, oxygen, and osteoblasts. An amorphous hard callus of irregularly arranged collagen and mineral, called woven bone, replaces the soft callus (Figure 6C). Lamellar bone gradually replaces woven bone during the remodeling process (Figure 6D), and the normal strength and structure of the skeletal element returns.

Cytokines

Scores of cytokines regulate the complex process of fracture healing [17]. Cytokines are molecular messengers made by one cell to communicate with other cells. Cytokines instruct other cells as to what to do, when to do it, and how much of it to do. The word cytokine comes from the Greek “cyto” meaning cell and “kinos” meaning movement, conveying the idea that they make cells move either physically or metabolically. The five different classes of cytokines are chemokines, interferons, interleukins, lymphokines, and tumor necrosis factors (TNFs) (Appendix 2). The five families have numerous subtypes that function in different tissues [18].

Cytokines are small proteins or peptides that cannot cross cell membranes. They work by binding to cell membrane receptors, changing the shape of the receptor, and causing the production of intracellular signaling molecules that go to the nucleus and influence gene activity. During fracture healing, cytokines upregulate genes facilitating cellular proliferation, differentiation, and synthetic biochemical reactions. 

Osteoblastogenic Cytokines

In 1965, orthopedic surgeon Marshall R Urist (1914-2001) discovered the osteoblastogenic cytokine bone morphogenetic protein (BMP). He implanted demineralized bone matrix into soft tissues, and ectopic bone formed [19]. He knew the substance was a protein because trypsin prevented bone formation. 

BMPs are members of the transforming growth factor (TGF-β) family of cytokines that have wide-ranging anabolic effects. Over 30 BMPs have been identified to date. All are glycoproteins of about 120 amino acids, and they bind to membrane receptors that phosphorylate intracellular SMAD proteins. Phosphorylated SMADs go to the nucleus to up-regulate genes for osteoinduction and osteoblast differentiation [20]. The greatest concentration of BMPs occurs during the hard callus phase of healing.

In 1980, Roeland Nusse and Harold Varmus discovered another osteoblastogenic cytokine called Wnt. They were investigating tumor viruses and discovered the presence of the gene int-1 that was the same gene that causes fruit flies to be Wingless. Wnt is a combination term from Wingless (W) and integration site-1 (nt). Wnts are 350 amino acid proteins that work by binding to cell membrane receptors called “Frizzled,” causing the release of β-catenin that goes to the nucleus, binds to transcription factors, and activates genes [21]. Nineteen Wnt proteins have been characterized so far. Mesenchymal stem cells, osteomacs, and osteoblasts synthesize and express Wnts that are crucial for fracture union and remodeling. 

Osteoclastogenic Cytokines

Two major osteoclastogenic cytokines are macrophage colony-stimulating factor (M-CSF) and receptor activator of nuclear factor κB ligand (RANKL) [22,23]. M-CSF is a 522 amino acid protein that stimulates hematopoietic precursor cells to differentiate into monocytes, macrophages, and ultimately osteoclasts. Another protein called c-Fms binds M-CSF and up-regulates genes that favor survival and proliferation of the cell. Both osteoblasts and osteocytes synthesize and express M-CSF. 

Osteoclasts require another cytokine for proper functioning, RANKL. The quest to understand RANKL came from the search for drugs to treat osteoporosis, eventually leading to the blockbuster drug denosumab. Investigators had identified a protein expressed by T and B lymphocytes called RANKL that turned out to be the same protein required by osteoclasts. RANKL, a 317 amino acid protein, is a member of the TNF family of cytokines. The main source of RANKL, like M-CSF, is from osteoblasts and osteocytes. RANK is the receptor protein present in osteoclast cell membranes that binds RANKL. This binding results in the formation of the intracellular signaling factor NF-κB that goes to the nucleus and up-regulates a variety of osteoclastic genes. Osteoblasts also make osteoprotegerin (OPG), a protein that inactivates RANKL. The RANKL/OPG ratio in the extracellular environment is a major regulator of bone remodeling. The more RANKL, the more bone resorption; the more OPG, the less bone resorption [24].

Hormones Versus Cytokines

Hormones and cytokines are both signaling molecules, but they differ in their distance of action. Hormones, like parathyroid hormone, are synthesized by glands and secreted into the general circulation where they can travel for a long distance, that is from a cell’s perspective, to reach their target cells in bone and kidney. Hormones are endocrine signals, and many are necessary for fracture healing like calcitonin, thyroxin, vitamin D, and glucocorticoids. 

Cytokines, on the other hand, are made by cells and act on other cells in a very close range. Cytokines can be released into the extracellular fluid, or they can be inserted directly into their own cell membranes to interact with adjacent cells. Cytokines are known as paracrine signals if secreted into the extracellular fluid, or autocrine signals if deployed within their own cell membrane.

Cells

Dozens of cell types contribute to the fracture healing process, but the three most predominant cells involved in the process are osteoblasts, osteoclasts, and osteocytes.

Osteoblasts

Osteoblasts are cuboid-shaped cells that are packed with mitochondria, endoplasmic reticulum, and Golgi apparatus, reflecting the massive amounts of energy needed to make the matrix proteins that comprise new bone tissue. They originate from pluripotent mesenchymal stem cells [25]. BMPs and Wnt upregulate the gene Runx2 which is a “master switch” controlling genes involved in osteoblast differentiation and function [26]. 

Osteoblasts synthesize and secrete an extracellular matrix called osteoid, composed mainly of type I collagen with smaller amounts of osteocalcin, osteopontin, fibronectin, elastin, laminin, proteoglycans, and glycosaminoglycans. Osteoblasts create an alkaline microenvironment favoring the reaction of phosphates with calcium to form hydroxyapatite, Ca10(PO4)6(OH)2, that precipitates out of the extracellular fluid and into the osteoid matrix. Osteoblasts have three possible fates. They can undergo apoptosis and disappear. They can become lining cells on the endosteal surface, or they can become osteocytes and reside within Haversian systems in cortical and cancellous bone tissue. 

Osteoclasts

Osteoclasts are cells that can completely dissolve bone tissue. They originate in the bone marrow from monocyte-macrophage precursors. Many of these cells can fuse to become a giant multi-nucleated osteoclast [27]. Multiple nuclei within the giant osteoclasts permit them to make ample amounts of messenger RNA necessary to direct the synthesis of large amounts of the enzymes needed to dissolve bone. Osteoclasts anchor to the bone surface using podosomes containing proteins known as integrins that adhere to bone matrix protein osteopontin. Within the osteoclast, an internal actin ring, looking somewhat like a hula hoop, is deployed around the cell’s perimeter and permits the osteoclast to seal and isolate the underlying bone tissue. This is important because osteoclasts release a cocktail of hydrochloric acid and degradative enzymes such as metalloproteinases, cathepsin K, and acid phosphatases that would be destructive to other areas if not contained. Finger-like projections extend from the undersurface of the osteoclast, the so-called ruffled border, which releases the acidic cocktail by the process of exocytosis. An osteoclast makes and resides in a microscopic pit called a Howship’s lacunae, first described by the English surgeon John Howship (1781-1841). Osteoclasts resorb the degraded osteoid and minerals by endocytosis and return the residual molecules to the extracellular fluid and hence to the general circulation. M-CSF1 initiates osteoclast differentiation, and RANKL keeps osteoclasts up and running via NF-κB that up-regulates the genes producing the acid and the enzymes.

Osteocytes

Osteocytes account for ~95% of all bone cells, and they have a long half-life of ~25 years, if they do not get caught up in a fracture or replaced during a bone remodeling cycle [28]. Osteocytes originate from osteoblasts that become embedded in their own mineralized osteoid. Osteocytes send out cell membrane extensions called dendrites in a three-dimensional network throughout the osteoid matrix and within little canals, canaliculi, to form gap junctions with other osteocytes and with a capillary in the central canal of the Haversian system. The fluid-filled lacunocanalicular system and a large number of gap junctions permit deeply embedded osteocytes to communicate almost instantaneously with the surface of the bone and with the circulation, as discussed above concerning mechanotransduction. 

Osteocytes use cytokines to regulate the balance between bone resorption and bone formation by expressing RANKL to stimulate osteoclasts and osteoprotegerin to inhibit osteoclasts. Osteocytes are the primary responders to mechanical stress and to biochemical imbalances. When parathyroid hormone binds to membrane receptors, osteocytes express RANKL, up-regulating osteoclasts, and elevating serum calcium. When vitamin D binds to osteocytes, they release fibroblast growth factor 23 (FGF23) which decreases phosphate resorption in the kidney and helps to control the serum phosphate level. 

Fracture union and remodeling require the interactions of scores of cytokines and dozens of cells. The cells synthesize and secrete matrix and engage in “crosstalk,” using the language of cytokines, M-CSF, RANKL, OPG, BMPs, and Wnt. Other cytokines like notch, Indian hedgehog, and platelet-derived growth factor play a key but transient role. Remodeling begins early and rapidly when osteomacs remove excess granulation tissue from the fibrovascular-soft callus (Figure 7A), but the process slows down after formation of the hard callus in which osteoblasts and osteoclasts predominate (Figure 7B). Remodeling becomes more mechanically sensitive toward the latter phases of remodeling as lamellar bone replaces the woven bone that was made for early stability and strength (Figure 7C). 

The periosteum is very important for fracture healing. A child’s periosteum is tough, flexible, and an important source of progenitor cells. The periosteum is held in place by Sharpey’s fibers which are aggregates of type 1 collagen that penetrate the bone surface and embed within the osteoid matrix. They were first described in 1846 by William Sharpey (1802-1880), a Scottish surgeon. The periosteum has three layers, a thin inner cambium layer, a thicker middle layer of cells, collagen, elastin, nerve fibers, and capillaries, and an outer tougher layer of fibroblasts and collagen [29]. After a fracture, the periosteum thickens due to hyperemia and cell proliferation. Progenitor cells preferentially initiate osteogenesis in the concavity of the fracture (Figure 8A). After the fracture becomes stable (Figure 8B), the periosteum continues to supply osteoblasts to reinforce the concavity while osteoclasts work under the periosteum on the convex surface to dissolve mechanically unfavorable bone (Figure 8C).

Bone remodeling, on the other hand, is a slow, life-long process that maintains and preserves bone strength and integrity. In 1963, Harold Frost showed that a team of cells called the basic multicellular unit (BMU) continuously removes and forms bone tissue in a “tightly coupled” process. The BMU team members include osteoclasts, osteoblasts, and endothelial cells [30]. New bone gradually replaces damaged and mechanically inferior bone, and in this way the BMUs repair microdamage and adjust to overall stress patterns experienced by bone tissue. This process removes and replaces about 10% of the adult skeleton each year.

Key concepts of pediatric fracture remodeling

The key concepts of pediatric fracture remodeling are as follows: (i) Cellular Piezo1 mechanoreceptors convert mechanical strains into intracellular chemical signals; (ii) Cytokines facilitate cellular communication during both the healing and remodeling process; (iii) BMP and Wnt are the major osteogenic cytokines, and M-CSF and RANKL are the major osteoclastic cytokines; (iv) The big three cells that dominate the process are osteoclasts, osteoblasts, and osteocytes.

## Conclusions

What is the answer to the question, how does the bone do it? The answer is that fracture union and remodeling are complex mechanobiological processes in which the cells respond to cytokine and mechanical signals to make new bone in areas of high stress and remove bone in areas of low stress, and under the influence of the mechanical stresses of daily living, such as gravity, muscle forces, acceleration and deceleration of the body and the limb, and within the constraints of systemic signals and homeotic genes, bone tissue is replaced and removed until the pre-injury strength and structure have been restored. In summary, pediatric fracture remodeling demonstrates the physiological principle, form follows function, i.e., Wolff's Law.

## Appendices

Appendix 1

Glossary of Mechanical Terms

Anisotropic: A substance with different physical properties when measured in different directions.  For instance, both bone and wood are stronger in compression along their longitudinal axis. Anisotropic substances are piezoelectric.

Basic multicellular units (BMUs): BMUs are a team of cells that dissolve bone and then fill the gap with new bone.

Fairbairn crane:  A type of crane with a curved jib patented in 1850 by the Scottish civil engineer Sir William Fairbairn (1789-1874).  Karl Culmann realized proximal femoral trabecular patterns resembled his stress calculations within Fairbairn’s crane. 

Mechanoreceptors: Cell membrane ion channels like Piezo1 and Piezo2 that open with pressure and permit ions flow causing intracellular biochemical changes.

Mechanotransduction: The process where mechanical stress is converted (transduced) into an intracellular signal.

Mechanostat theory: A theory proposed by Harold Frost to explain bone remodeling.  A physiological mechanism monitors overall mechanical loading on bone, and if the loads are within the physiological range, then bone mass is maintained, but when bone experiences underloading or overloading then bone remodeling takes place. 

Piezoelectricity: Electric polarization of a material caused by application of mechanical stress. 

Piezo1 and Piezo2: Transmembrane proteins that respond to mechanical stimuli.  Piezo1 is the major mechanoreceptor in chondrocytes, osteoblasts, osteoclasts, and osteocytes, that responds to shear, pressure, and stretch.  Piezo2 is involved in proprioception.  The corresponding genes are PIEZO1 and PIEZO2.

Sharpey’s fibers: Collagenous fibers anchoring the periosteum to bone, described in 1846 by William Sharpey (1802-1880) a Scottish surgeon, anatomist, and physiologist. 

Strain: Deformation or displacement of a structure caused by applied force.

Stress: A force applied to a structure.

Wolff’s Law: A statement that the shape and strength of a bone (form) is the result of forces experienced by the bone (function).

Appendix 2

Glossary of Bone Biology Terms

Alkaline phosphatase:  ALP is an enzyme attached to cell membrane of osteoblasts that works best a pH of 8.5 or higher. The enzyme is a regulator of bone mineralization by supplying phosphate for hydroxyapatite formation by hydrolyzing inorganic and organic phosphate compounds.  Used as a biomarker for bone formation.

Apoptosis: A series of biochemical pathways in cells, that once activated, results in death of the cell.

Bisphosphonates: Compounds like alendronate that inactivate GTPase enzymes in osteoclasts, preventing formation of the sealing zone and causing osteoclast apoptosis.

Bone morphogenetic proteins (BMPs): BMPs are multifunctional cytokines belonging to the TGF β superfamily. Glycoproteins of ~120 amino acids long, they are important to fracture healing by stimulating stem cell proliferation and differentiation.

Basic multicellular units: BMUs are a team of cells, including osteoclasts, osteoblasts, and endothelial cells that remove bone and make new bone to fill the area. 

Chemokines: A subgroup of cytokines that recruit mesenchymal cells, monocytes and macrophages to migrate into the area of their presence.

Cytokines: Small signaling proteins that are expressed by or released by one cell to communicate with other cells by binding to surface receptors and causing biochemical changes in the receptor cell. 

Endosteum: The inner surface and lining of a long bone.

Homeotic genes: Master regulator genes that direct overall body pattern or body parts that are in specific areas.

Interferons: A subgroup of cytokines made by white blood cells that help mobilize the immune response to pathogens.

Interleukins: A group of more than 50 cytokines produced by leukocytes and acting to promote the development of T and B lymphocytes.  Interleukin-6 (IL-6) appears in the hematoma about one day after a fracture.

Ligand: A chemical signal (usually a small protein) released by one cell that binds to another cell.

Lymphokines: A subgroup of cytokines produced by T cells that attract other immune cells.

Macrophage colony-stimulating factor:  M-CSF, also known as colony-stimulating factor, is a cytokine that controls the proliferation, differentiation, and activation of mesenchymal cells to differentiate into macrophages and osteoclasts.

Matrix metalloproteinases (MMPs): MMPs are a family of at least 50 endopeptidases containing a zinc atom (the metallo part) that degrade extracellular matrix proteins including collagen.  They are regulated by other proteins called TIMPs, tissue inhibitors of metalloproteinases.  Osteoclasts secrete mostly MMP-9.

Osteocalcin: A protein that is high in the amino acid gamma-carboxyglutamic acid, secreted by osteoblasts, that tightly binds calcium, and can be used as a biochemical marker for bone formation.

Osteonectin: A glycoprotein secreted by osteoblasts that initiates mineralization by tightly binding calcium to type I collagen.

Osteopontin: A heavily phosphorylated structural protein in mineralized matrix made by osteoblasts and osteocytes. Osteoclasts form sealing zones by binding to osteopontins.

Osteoclastogenesis: The formation and proliferation of osteoclasts by fusion of hematopoietic monocytic precursor cells.  The process is stimulated by the cytokines M-CSF and RANKL.

Osteoid: Meaning “bone like.” osteoid is unmineralized bone matrix. Consisting mostly of type I collagen, but also non-collagen proteins like osteopontin, osteonectin, proteoglycans, and glycosaminoglycans.

Osteoprotegerin: OPG is a protein produced by osteoblasts that binds RANKL, prevents osteoclast stimulation and decreases bone resorption.

Osteomacs: A subtype of macrophages that reside on the periosteal and endosteal surface of bone.  They have been shown to be present in much as 15% of all marrow cells. 

RANKL: Receptor activator of nuclear factor kappa-light-chain-enhancer of activated B cells ligand is a protein of the TNF cytokine superfamily. Originally discovered as a regulator within the immune system, as the name implies, NF-κB controls DNA transcription, and RANKL stimulates osteoclastogenesis. 

Sclerostin: A glycoprotein expressed by osteocytes that decreases bone formation by inhibiting Wnt and bone morphogenetic proteins and upregulating RANKL expression. 

SMAD proteins: Signal transducers for TGF-β ligands that when activated in the cytoplasm go to the nucleus to activate genes involved in skeletal development and repair.

Transcription factors: A wide variety of proteins that attach to specific regions of DNA to control expression of genes. They initiate and control the transcription of DNA segments into messenger RNA that exits the nucleus to bind with ribosomes and synthesize proteins.

Transforming growth factor β: TGF βs are a family of many similar peptides that bind to cell receptors and trigger intracellular signals causing cell proliferation and maturation (differentiation). TGF β-1 regulates bone and cartilage formation.

Transcriptomes: Combination term from transcription and genome, refers to all the types of messenger RNA synthesized by a cell. 

Tumor necrosis factors: TNFs are proinflammatory and antineoplastic cytokines produced by macrophages and lymphocytes that stimulate osteoclastogenesis.

Wnt: Wnts are peptides that control cell proliferation during bone development, modeling, and remodeling by upregulating osteogenic genes.  Wnt binds to a membrane receptor called Frizzled (a transmembrane G protein-coupled receptor) that activates another protein called disheveled which inhibits the enzyme glycogen synthase kinase 3β resulting in the buildup of unphosphorylated β-catenin that goes to the nucleus to activate (upregulate) osteogenic genes.
